# Sub-Antimicrobial Dosage Scheme of Doxycycline for the Chronic Treatment of Bronchiectasis in a Dog

**DOI:** 10.3390/vetsci9030137

**Published:** 2022-03-15

**Authors:** Viktor Szatmári, Ingeborg M. van Geijlswijk

**Affiliations:** 1 Department of Clinical Sciences, Faculty of Veterinary Medicine, Utrecht University, 3584 CM Utrecht, The Netherlands; 2 Department of Population Health Sciences, Faculty of Veterinary Medicine, Utrecht University, 3584 CM Utrecht, The Netherlands; i.m.vangeijlswijk@uu.nl

**Keywords:** antibiotics, matrix metalloproteinase, bacterial pneumonia, prevention

## Abstract

A 9-month-old German shepherd dog was examined because of a chronic cough, exercise intolerance and labored breathing, as well as recurrent episodes of lethargy with anorexia. Multifocal severe bronchiectasis and neutrophilic bronchitis was found with thoracic computed tomography and cytology of bronchoalveolar lavage fluid, respectively. While oral azithromycin was administered, clinical signs were absent. However, stopping azithromycin lead repeatedly to presumed bacterial pneumonia within 1–2 months. With sub-antimicrobial dosed oral doxycycline (initially 1.5 mg/kg once daily for 3 months, then 0.7–0.5 mg/kg once daily for 6 months), the dog remained free from clinical signs. Bronchiectasis is characterized by marked irreversible bronchial dilation. Accumulation of intraluminal mucopurulent material and neutrophilic inflammation cause chronic cough and recurrent bacterial pneumonia. For therapy, life-long oral antibiotics are recommended. Chronic antibiotic administration, however, can select resistant bacterial strains. Though both azithromycin and doxycycline possess anti-inflammatory effects, doxycycline has these off-target properties at a sub-antimicrobial dose. In this report, a chronic sub-antimicrobial dose of doxycycline resulted in the resolution of chronic cough, exercise intolerance and labored breathing, and prevented recurrence of suspected bacterial pneumonia in the long-term in a dog with severe bronchiectasis. Beneficial effect of doxycycline is probably related to its anti-inflammatory effects rather than its antimicrobial properties.

## 1. Introduction

Bronchiectasis is defined as a severe, irreversible dilatation of bronchi in a single or several lung lobes with secondary accumulation of mucopurulent intraluminal material [1,2,3,4,5,6]. The condition can have many underlying etiologies, such as primary ciliary dyskinesia, chronic infectious or non-infectious inflammatory disorders of endogenous or exogenous origin, and it can also be idiopathic [1,2,3,4,5,6]. The leading clinical sign of bronchiectasis is a chronic cough, which is caused by the accumulation of a large amount of mucopurulent material in the dilated bronchi. In addition, dogs with bronchiectasis can also suffer from recurrent episodes of bacterial pneumonia [3]. If bronchiectasis is localized to a single lung lobe, surgical lobectomy can be curative [3]. However, if bronchiectasis affects several lung lobes, the mainstem therapy is lifelong, continuous or intermittent, oral antibiotic therapy [3]. Antibiotic administration has two aims: (1) to prevent secondary bacterial pneumonia and (2) to control respiratory signs directly related to bronchiectasis [3,6]. Azithromycin is often the first choice of antibiotic agent for bronchiectasis in human and veterinary medicine [3,7].

Our case report shows for the first time that sub-antimicrobial dose of doxycycline can successfully control clinical signs of bronchiectasis and prevent recurrent bacterial pneumonia on long term in a dog, due to its suspected off-target anti-inflammatory and immunomodulatory effects [8].

## 2. Case Presentation

A 9-months-old intact male German shepherd dog weighing 28 kg was presented to the Cardiology and Pulmonology service of the authors’ teaching hospital, Companion Animal Clinic of the Utrecht University in May 2020 because of a chronic cough, exercise intolerance, and labored breathing for a duration of at least 2 months, as long as the present owner had owned the dog. No history was available regarding the first 7 months of the dog’s life, besides that it was confiscated from the previous owner because of neglection. The dog had been coughing several times a day, more often with exercise, at times productive. Exercise intolerance manifested as laying down after 5 min of running or playing, but it could walk for 40 min with ease. No vomiting, regurgitation, sneezing, nasal or ocular discharge had ever been noticed. The dog’s appetite had been suboptimal, and the stool had been intermittently soft, while the frequency of defecation and the amount of feces remained unchanged.

The initial presenting complaints to the referring veterinarian were anorexia, lethargy, exercise intolerance, cough and dyspnea. The referring veterinarian found increased rectal temperature (39.7 °C) and on thoracic radiographs a consolidated right middle lung lobe. A tentative diagnosis of bacterial pneumonia was made. A course of oral amoxicillin and clavulanic acid (13.4 mg/kg BID for 2 weeks) was prescribed, which resolved the anorexia and the fever, but not the cough, exercise intolerance and the labored breathing.

At presentation to the authors’ clinic, physical examination revealed a bright, alert, and responsive dog with a body condition score of 3 out of 9. A mildly increased inspiratory effort was noted, which worsened with stress and exercise. The respiratory rate was 48 breaths per minute, the femoral pulses were strong and regular with a frequency of 116 beats per minute, and the mucous membranes were pink with a capillary refill time of 1 s. Rectal temperature was 38.8 °C. Lung auscultation revealed bilaterally increased inspiratory lung sounds. Tracheal palpation elicited a soft, moist, nonproductive cough.

Initial diagnostic tests consisted of thoracic radiographs, blood test and fecal parasitological examination. Radiographs showed an interstitial to alveolar unstructured soft tissue opacity in all right-sided lung lobes, while the right cranial and right caudal lung lobes were increased in size. In the right cranial lung lobe, a soft tissue nodule of about 1 cm was present. The ventral part of the right middle lung lobe was completely consolidated with vesicular soft tissue opacity. A homogenous alveolar soft tissue opacity was present in the left cranial lung lobe. Due to a decreased volume of the left cranial lung lobe, the heart was shifted to the left and made direct contact with the chest wall. The volume of the left caudal lung lobe was also decreased. Severely dilated bronchi were visible to the periphery in the right middle and left cranial lung lobes. In addition, a mild amount of pleural effusion was suspected. Hematology showed a mild leukocytosis (21.8 × 10^9^/L, reference range 4.5–14.6 × 10^9^/L) due to a mild neutrophilia (12.6 × 10^9^/L, reference range 2.9–11.0 × 10^9^/L), a mild lymphocytosis (5.8 × 10^9^/L, reference range 0.8–4.7 × 10^9^/L), a mild monocytosis (1.4 × 10^9^/L, reference range 0.0–0.9 × 10^9^/L) and a mild eosinophilia (2.0 × 10^9^/L, reference range 0.0–1.6 × 10^9^/L). Biochemistry was unremarkable and antigen tests for *Dirofilaria immitis* and *Angiostrongylus vasorum* were negative. Fecal flotation revealed cysts of *Giardia intestinalis*, while Baermann larva isolation was negative of a 3-day mixed stool sample.

**Figure 1:**
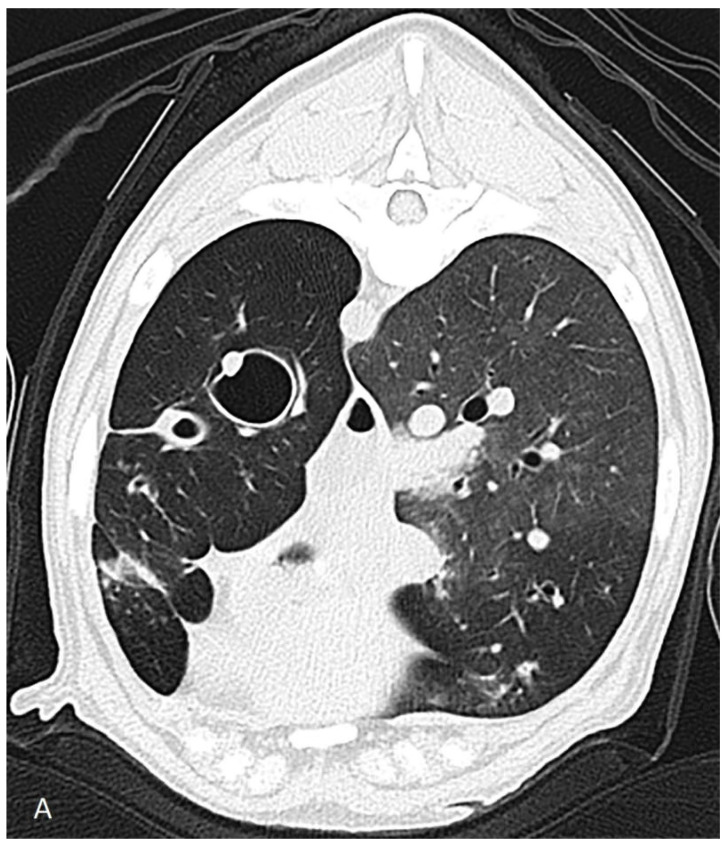

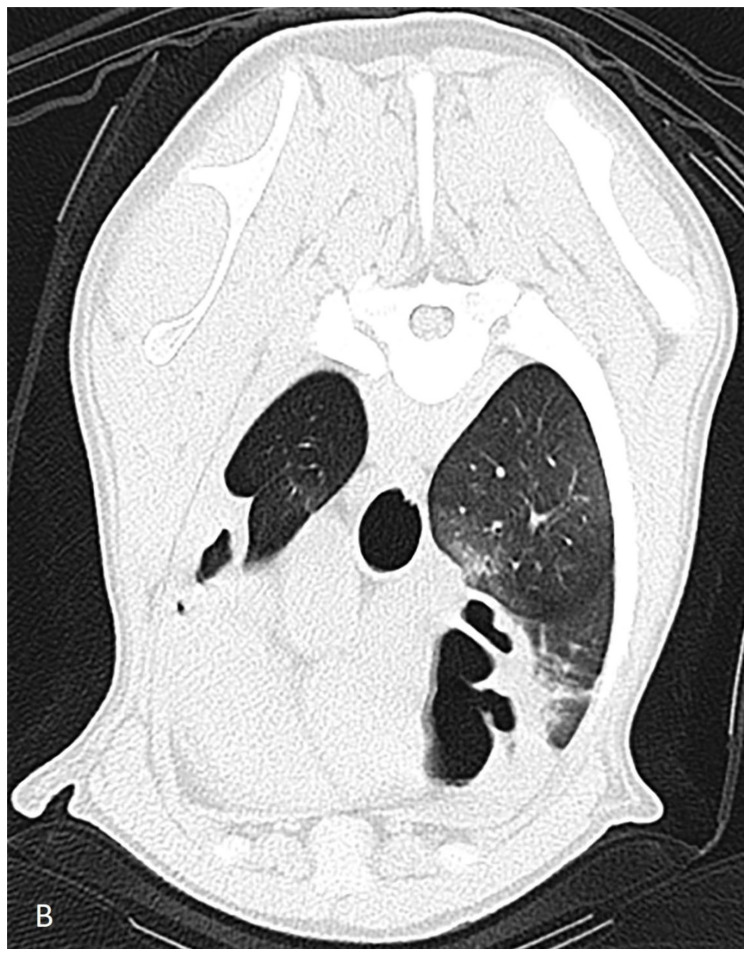
Computed tomographic scan of a 9-month-old German shepherd dog with multifocal bronchiectasis of unknown origin. Left of the animal is on the left side of the image. (**A**) Severe dilation of the lobal bronchus in the center of the left caudal lung lobe can be appreciated. The right caudal lung lobe has a larger volume compared to that of the left one and has normal tapering bronchi to the periphery. (**B**) Severe saccular dilation and lack of tapering to the periphery of the lobal bronchus of the right cranial lung lobe can be appreciated.

The dog was hospitalized and the next day a computed tomography scan, followed by a bronchoscopy and a bronchoalveolar lavage, were performed under general anesthesia. Computed tomography revealed severe dilation of the lobal bronchi of the left cranial, left caudal, right cranial, right middle and right accessory lung lobes (Figure 1). The rest of the bronchi showed thickened bronchial walls. The right sided lung lobes had a larger volume compared to those of the left, but the accessory lung lobe and the left cranial lung lobes were not aerated. Several poorly circumscribed interstitial to alveolar regions were noticed in the whole lung field in the peribronchial regions, most prominently in the right cranial lung lobe. The radiographically noticed soft tissue nodule was not recognized.

Bronchoscopy, performed with a rigid optic after extubation, revealed a moderate amount of mucopurulent material in the pharynx, in the tracheal lumen and in the lumen of the lobar bronchus of the accessory lung lobe. The left and right principial bronchi and the lobar bronchi of the right and left caudal lung lobes showed, besides mild diffuse mucosal hyperemia, no abnormalities. The mucopurulent material of the accessory lung lobe was removed with suction and it was collected in a sterile syringe. In addition, a bronchoalveolar lavage was performed from the right and the left caudal lung lobes using a physiologic NaCl solution of 40 °C with two aliquots of 12 mL. The samples were submitted to cytologic examination as well as bacteriologic and fungal culture. Recovery from general anesthesia was uneventful. Cytology of the undiluted exudate gained from the accessory lobe revealed a large number of neutrophilic granulocytes, and a few eosinophilic granulocytes and macrophages, without visible bacteria. Bronchoalveolar lavage fluid of the right and left caudal lung lobes were processed to cytologic examination after cytospin. Cytology revealed many neutrophilic granulocytes and a low number of ciliary epithelial cells, without visible bacteria. Bacterial and fungal culture of the pure sample from the accessory lung lobe and the mixed sample of the bronchoalveolar lavage fluid from the right and left caudal lung lobes revealed no fungi, but a sporadic bacterial growth of five different bacteria. The diagnosis of idiopathic multifocal bronchiectasis with chronic neutrophilic bronchitis was made. Multifocal bronchiectasis is an irreversible, incurable condition. The results of the bacterial culture was considered a contamination.

Oral azithromycin was prescribed (18 mg/kg once daily) for a week, followed by twice a week administration (18 mg/kg per occasion) for 3 months with the approval of the institution’s antibiotic advisory board (A-team). Two months later, a re-check examination took place. The owner reported that the dog had stopped coughing within a week, his endurance had markedly improved, and no episode of lethargy with anorexia had appeared. The sleeping respiratory rate was always below 30 breaths/min. On the days when azithromycin was administered the dog had anorexia. Physical examination findings were unremarkable. The dog lost 1 kg bodyweight compared to 2 months prior. Thoracic radiographs were repeated and showed mild decrease of the interstitial to alveolar pulmonary changes. The bronchiectasis and consolidation of lung lobes were identical to those of two months earlier.

Azithromycin therapy was stopped 1 month after the re-check. The dog continued to show no clinical signs for about 8 weeks. Thereafter, sudden onset of cough, dyspnea, lethargy, exercise intolerance, anorexia and fever appeared. These findings were interpreted by the referring veterinarian as a bacterial pneumonia. Oral azithromycin was prescribed again with the same dosage as previously, i.e., after a weeklong once daily administration, twice-a-week regime for 3.5 months. The clinical signs resolved. While the dog was receiving azithromycin, the referring veterinarian performed a surgical triple pelvic osteotomy on the left side because of a severe hip dysplasia diagnosed previously on radiographs. One month after stopping the azithromycin therapy, a sudden onset of cough, dyspnea, lethargy, exercise intolerance and anorexia reappeared. The referring veterinarian prescribed azithromycin for a week and referred the dog again to the authors’ institution. In the meantime, the clinical signs resolved.

At presentation to the authors’ institution, the physical examination findings were unremarkable. The body condition score was 4 out of 9, and the bodyweight was 33 kg. According to the owner, the dog had not coughed, had a good appetite and it could walk for 1.5 h easily. The institution’s A-team recommended a sub-antimicrobial dose of oral doxycycline (1.5 mg/kg, once daily) for the long-term therapy instead of azithromycin. This decision was made to reduce the chance for selecting azithromycin-resistant bacterial strains. The owner, 3 months later, reported via email that the dog was free of clinical signs. The daily doxycycline dose was then halved to 0.7 mg/kg once daily. This decision was made to bring the dose closer to reported recommended human dosage [9]. After this, 5 months later, the owner reported via email that the dog continued to have no clinical signs and the dog gained weight (bodyweight of 44 kg), which resulted in a further reduction of the doxycycline dose to 0.5 mg/kg once daily. No follow up radiographs were performed because (1) bronchiectasis is an irreversible disease and (2) radiographic changes showed no correlation with the presence or absence of clinical signs based on the previously made radiographs. Follow-up-computed tomographic scan was considered unethical because of the potential risks and inconvenience of general anesthesia for the dog and the high costs for the owners, whereas the results would not affect the therapeutic plan.

## 3. Discussion

Though azithromycin solved the clinical signs of bronchiectasis and prevented recurrent bacterial pneumonia in the presented case, it also resulted in anorexia of short duration on the day of administration, which is a reported adverse reaction of this medication (as written in the specification of medicinal product characteristics, SmPC). Contrarily, doxycycline did not cause any side effects.

Based on the present case report, we suspect that the anti-inflammatory properties of the doxycycline were sufficient to control clinical signs of bronchiectasis and prevent recurrent bacterial pneumonia. The mechanism of this effect might be the reduction of bronchial mucus secretion and the neutrophilic granulocyte infiltration of the bronchi [7,9]. Therefore, antibiotic therapy would only be indicated in an event where the dog is presented with clinical signs of suspected bacterial pneumonia (i.e., in the present case anorexia, cough and fever). In case of suspected pneumonia, the dose of oral doxycycline can be increased to the antimicrobial dose of 5–10 mg/kg once or twice a day until cessation of the acute clinical signs. The most likely reason why therapy with amoxicillin and clavulanic acid prescribed by the referring veterinarian did not result in resolution of chronic chough is that beta lactam antibiotics are not known to have off-target anti-inflammatory and immunomodulatory effects. Because chronic cough in the case of bronchiectasis is thought to be related to increased mucus secretion and nonbacterial neutrophilic bronchial wall inflammation, pure antibiotic therapy could result in insufficient clinical improvement of respiratory signs. On the other hand, amoxicillin and clavulanic acid did solve the acute clinical signs of anorexia and fever, which were probably the result of a bacterial pneumonia.

Since the anti-inflammatory dose range of doxycycline has yet to be determined in dogs, we chose an initial dose close to a reported canine study on periodontal condition [8]. Because the longest disease-free period without antibiotic therapy in the presented dog was 2 months, we administered doxycycline in this dosage for 3 months to test its long-term efficacy. Afterwards, the dose was reduced to 0.7 mg/kg once daily, which is close to the dose used in humans [9]. As a comparison, the anti-inflammatory dose of doxycycline in humans is 20 mg BID, which is equivalent to 0.5 mg/kg/day in the case of an 80 kg person [9]. Because of the weight gain of the dog and unchanged daily administration of doxycycline, the dose gradually decreased to 0.5 mg/kg once daily, which seemed to be equally effective in controlling clinical signs on long term.

Limitation of the present case report is that the lowest effective dose of doxycycline was not determined by further reduction of the daily dose. Neither was the therapy interrupted to see how quick the clinical signs would recur. Finally, the underlying pathology that lead to the bronchiectasis in the present dog was not identified.

In humans, azithromycin is the first choice of antibiotics for long term treatment of bronchiectasis [1,6]. This macrolide antibiotic possesses not only antimicrobial properties, but also simultaneous anti-inflammatory effect [1,6,10,11,12]. Because published clinical trials on therapy in dogs with bronchiectasis are lacking, recommendations from veterinary textbooks and human studies are generally followed. Both sources recommend azithromycin for chronic use in bronchiectasis [3,6,13]. The World Health Organization (WHO) declared azithromycin as a “critically important and highest priority antimicrobial for human medicine” [14]. Because chronic antibiotic use will result in selection of resistant bacteria strains, azithromycin could better be reserved for infections where no alternative therapy is available based on antimicrobial sensitivity testing [15].

Doxycycline is a tetracycline antibiotic, and its use is widespread in veterinary medicine as a first line antimicrobial agent in suspected pneumonias, even in the absence of bacterial culture of airway samples [3]. Similar to azithromycin, doxycycline possesses anti-inflammatory and immunomodulatory effects [16,17,18]. The major advantage of doxycycline compared to azithromycin is that these off-target effects are present in dosages below the usual antimicrobial dose, in so-called sub-antimicrobial doses [8,19]. In humans, the anti-inflammatory and immunomodulatory effects of doxycycline have been utilized in a number of chronic inflammatory disorders, among others, in dermatology, ophthalmology, pulmonology and dentistry [9,16,17]. In dogs, the anti-inflammatory effect of doxycycline has so far only been shown in periodontal disease, in a controlled clinical trial using a dose of 2 mg/kg once daily, as opposed to the antimicrobial therapeutic dose of 5–10 mg/kg once or twice daily [8]. Though the WHO-classification of doxycycline is lower than that of azithromycin, it still falls in the category of “highly important antimicrobials for human medicine” [14]. 

The mechanism of anti-inflammatory and immunomodulatory effect of doxycycline is related to the protection of extracellular matrix, including collagen. Matrix metalloproteinases are known to be the key regulators of tissue destruction and are known to degrade extracellular matrix of human airways [20,21,22]. In addition, matrix metalloproteinases act as chemotactic factors for neutrophilic granulocytes, inducing their local accumulation [21,23]. Neutrophil granulocytes secrete, among other substances, neutrophil elastase, whose chronic effects involve degradation of airway extracellular matrix and prevention of adequate tissue repair, resulting in abnormal airway remodelling [9,24]. As the only drug in human medicine, doxycycline is licensed by the United States Food and Drug administration (FDA) for inhibition of matrix metalloproteinase [9].

## 4. Conclusions

The present case report showed clinical efficacy of a sub-antimicrobial dose of doxycycline in the long-term management of bronchiectasis in a dog by resolving chronic clinical signs, such as cough, labored breathing and exercise intolerance, as well as by preventing recurrent bacterial pneumonia. This finding could result in reduction of antibiotic use in dogs with bronchiectasis. In addition, we suspect that the anti-inflammatory effect, and not the antimicrobial effect, of doxycycline had a key role in controlling airway inflammation in bronchiectasis.

## Data Availability

Not applicable.
